# Genome Sequence of *Lactobacillus gastricus* PS3, a Strain Isolated from Human Milk

**DOI:** 10.1128/genomeA.00489-13

**Published:** 2013-07-11

**Authors:** Virginia Martín, Nivia Cárdenas, Esther Jiménez, Antonio Maldonado, Juan Miguel Rodríguez, Leonides Fernández

**Affiliations:** Departamento de Nutrición, Bromatología y Tecnología de los Alimentos, Universidad Complutense de Madrid, Madrid, Spaina; Departamento de Biotecnología de Alimentos, Instituto de la Grasa, Consejo Superior de Investigaciones Científicas–CSIC, Seville, Spainb

## Abstract

*Lactobacillus gastricus* is a mostly unknown lactobacilli species associated with mucosal surfaces. We present the draft annotated genome sequence of *L. gastricus* strain PS3, isolated from a human milk sample, to provide new insights into its biology and to characterize those genes related to advantageous technological and beneficial properties.

## GENOME ANNOUNCEMENT

*Lactobacillus gastricus* was first isolated from gastric biopsy specimens from healthy humans and identified and described by Roos et al. (1, 2). This species has been associated with the *Lactobacillus reuteri* group according to its phylogenetic relatedness (3, 4). A recent investigation of breast milk from healthy women revealed the presence of *L. gastricus* strains in about one-third of 20 milk samples analyzed, together with other *Lactobacillus* species such as *Lactobacillus casei*, *Lactobacillus gasseri*, *Lactobacillus fermentum*, *Lactobacillus plantarum*, *L. reuteri*, *Lactobacillus salivarius*, and *Lactobacillus vaginalis* (5). The human milk microbiota has been regarded as essential for initiation and development of infant gut colonization (6–8). Furthermore, strains of *L. gasseri*, *L. fermentum*, and *L. salivarius* isolated from this biological fluid have demonstrated excellent probiotic potential (9, 10). After an initial characterization of technological and probiotic properties in a collection of *L. gastricus* strains isolated from human milk, strain PS3 was selected on the basis of some traits of probiotic and technological relevance and identified via 16S rRNA gene sequencing.

In order to get a deeper knowledge of the technological and probiotic properties of this strain, we performed whole-genome sequencing of *L. gastricus* PS3 by 454 pyrosequencing on a GS-FLX sequencer to 19.14-fold coverage (454 Life Sciences, Brandford, CT). The initial draft assembly generated 93 contigs using the Newbler program version 2.3 (Roche Applied Science). The draft genome of *L. gastricus* PS3 consists of 1,904,872 bases with an average GC content of 41.8% and contains a total of 1,386 protein-encoding sequences and 43 RNA-encoding sequences (40 tRNAs and 3 rRNAs). Coding regions were predicted using the BG7 system (Era7 Technologies, Granada, Spain), which proceeds from protein similarity detection to open reading frame (ORF) prediction and is tolerant of sequencing errors in start and stop codons, frameshifts, and assembly or scaffolding errors (11). The semiautomatic annotation of the sequences resulted in 80 final contigs, 1,269 protein-coding genes, 40 tRNA-encoding genes, and 3 rRNAs.

More than 20 peptidases and proteases and several peptide transporter genes were predicted. This highly complex proteolytic system would allow the growth in milk and compensate for deficiencies in amino acid biosynthesis. Significantly, genes encoding three different putative glutamate-cysteine ligases (PS3_6383, PS3_8988, and PS3_21018) that synthesize γ-glutamylcysteine were detected. This is the major low-molecular-weight thiol that protects against oxidative stress in some lactic acid bacteria (12). Also, one putative gene coding glutamate decarboxylase (PS3_14606) was identified. This enzyme catalyzes the synthesis of γ-aminobutyric acid, an amino acid that contributes to bacterial acid resistance and has potential as a bioactive compound in humans (13). Putative genes coding for transport systems and enzymes related to utilization of diverse carbohydrates such as fructose, galactitol, mannose, lactose, cellobiose, sucrose, and β-glucosides were also identified, as well as genes encoding two putative esterases and a GDSL lipolytic enzyme.

### Nucleotide sequence accession numbers.

The results of this whole-genome shotgun project have been deposited at DDBJ/EMBL/GenBank under the accession number AICN00000000. The version described in this paper is the first version, AICN01000000.
